# Risk factors for interval breast cancer: insights from a decade of a mammography screening program

**DOI:** 10.1007/s10549-025-07619-4

**Published:** 2025-02-12

**Authors:** Jonas Subelack, Rudolf Morant, Marcel Blum, Alena Eichenberger, Alexander Geissler, David Ehlig

**Affiliations:** 1 Chair of Health Economics, Policy and Management, School of Medicine, University of St.Gallen, St. Jakobstr. 21, 9000 St.Gallen, Switzerland; 2 Cancer League of Eastern Switzerland, St.Gallen, Switzerland

**Keywords:** Interval breast cancer, Interval cancer, Interval carcinoma, Breast cancer, Mammography screening program, Risk factors

## Abstract

**Purpose:**

Breast cancer remains a major global health issue, with mammography screening programs (MSPs) being critical for early detection to improve survival. Interval breast cancers (IBC) are an important quality criterion and have been linked with increased mortality. We aimed to identify risk factors for IBC diagnoses, based on MSP data.

**Methods:**

In this retrospective cohort study, we merged data from the Swiss MSP “donna” with data from cancer registries from 2010 to 2019 to categorize cases as IBC or screen-detected breast cancer (SBC). We compared the incidence, tumor characteristics, and survival proportions of women with IBC versus SBC. We used a multivariable Poisson regression with robust errors to identify risk factors for IBC diagnoses.

**Results:**

We identified 1134 breast cancer cases, specifically 251 IBC and 883 SBC. The 7-year survival proportions significantly deviated with 92.9% for women with IBC and 96.4% for women with SBC (*p* < 0.05). Women with IBC are diagnosed with significantly higher tumor stages (*p* < 0.05) and have a worse tumor biology in multiple dimensions e.g. larger tumor size or more often triple negative (*p* < 0.05). Higher breast density (BI-RADS d risk ratio (RR): 3.293), certain age groups (55–59 years RR: 1.345), and a family breast cancer history (RR: 1.299) were identified as significant (*p* < 0.05) risk factors for IBC diagnoses.

**Conclusions:**

Women with IBC had lower overall survival proportions than women with SBC, possibly due to higher stages at diagnosis. Increased breast density and a positive family history of breast cancer could encourage MSPs to personalize their screening process (e.g. additional diagnostics).

## Purpose

Breast cancer is a predominant health concern globally, with steadily rising incidence [1, 2]. The widespread adoption of mammography screening programs (MSPs) and technological advancements in treatment options have significantly reduced mortality and disease burden [3, 4]. Randomized controlled trials in the 1970’s and 1980’s demonstrated a reduction of breast carcinoma (BC)-related mortality for woman participating in an MSP [5]. The goal of an MSP is to detect BC at a favorable early stage, thus reducing the burden of disease through less aggressive treatments as well as improved survival proportions [5–11]. The incidence of interval breast carcinoma (IBC), where no BC has been identified during regular screening but is diagnosed outside the MSP within 24 months after screening, is an important quality criterion [12]. Unfortunately, IBCs are associated with more aggressive tumor characteristics, significantly higher mortality rates, and more invasive treatments [13, 14]. Therefore, it is important to identify risk factors available during screening that are associated with a higher likelihood of an IBC diagnosis, which could be used to improve and individualize MSPs.

Prior, a study has identified high breast density and younger age as risk factors for IBC [15]. Another study identified among others hormone replacement therapy as risk factor [16]. However, the findings are limited as they do not enable improved screening practices. Furthermore, to the best of our knowledge, no study has yet analyzed such a comprehensive combination of factors that were already available at screening, and no study of IBCs has been conducted within Switzerland so far. Therefore, our study addresses this research gap and identifies MSP-available risk factors for IBC diagnoses, based on a comprehensive Swiss dataset. This is of practical relevance to inform strategies for quality control and improvement in early BC detection as for example via risk factor-oriented, personalized screening intervals.

## Methods

### Study population

The study included all 50- to 69-year-old women who participated in the MSP in the Swiss cantons of St.Gallen and Grisons (covering around 10% of the Swiss population) between 2010 and 2019 and who were diagnosed with invasive or in situ BC (ICD10: C50 and D05). Cancer data were retrieved from the databases of the cancer registries of Eastern Switzerland and Grisons-Glarus, which document all cancer cases diagnosed in the two cantons, follow-up the vital status of each patient via the cantonal administrations and record the date of death or the date of last contact [17]. According to the Swiss national agency for cancer registration, the “results show the high overall quality of data held by cancer registries in Switzerland” [18].

### The MSP “donna” in the Swiss cantons of St.Gallen and Grisons

All women between 50 and 69 years of age in the cantons of St.Gallen (since 2010) and Grisons (since 2011) are invited every two years to voluntarily participate in the MSP, covered by the compulsory health insurance except for a small deductible. Screening is generally intended for women at a certain age group with an average risk of BC, and women at high risk (individual doctors’ assessment) can be provided with individualized solutions (e.g. supplemental imaging including breast MRI, shorter screening interval, initiation of screening at an earlier age).

### Sample generation and data collection

**Fig. 1:**
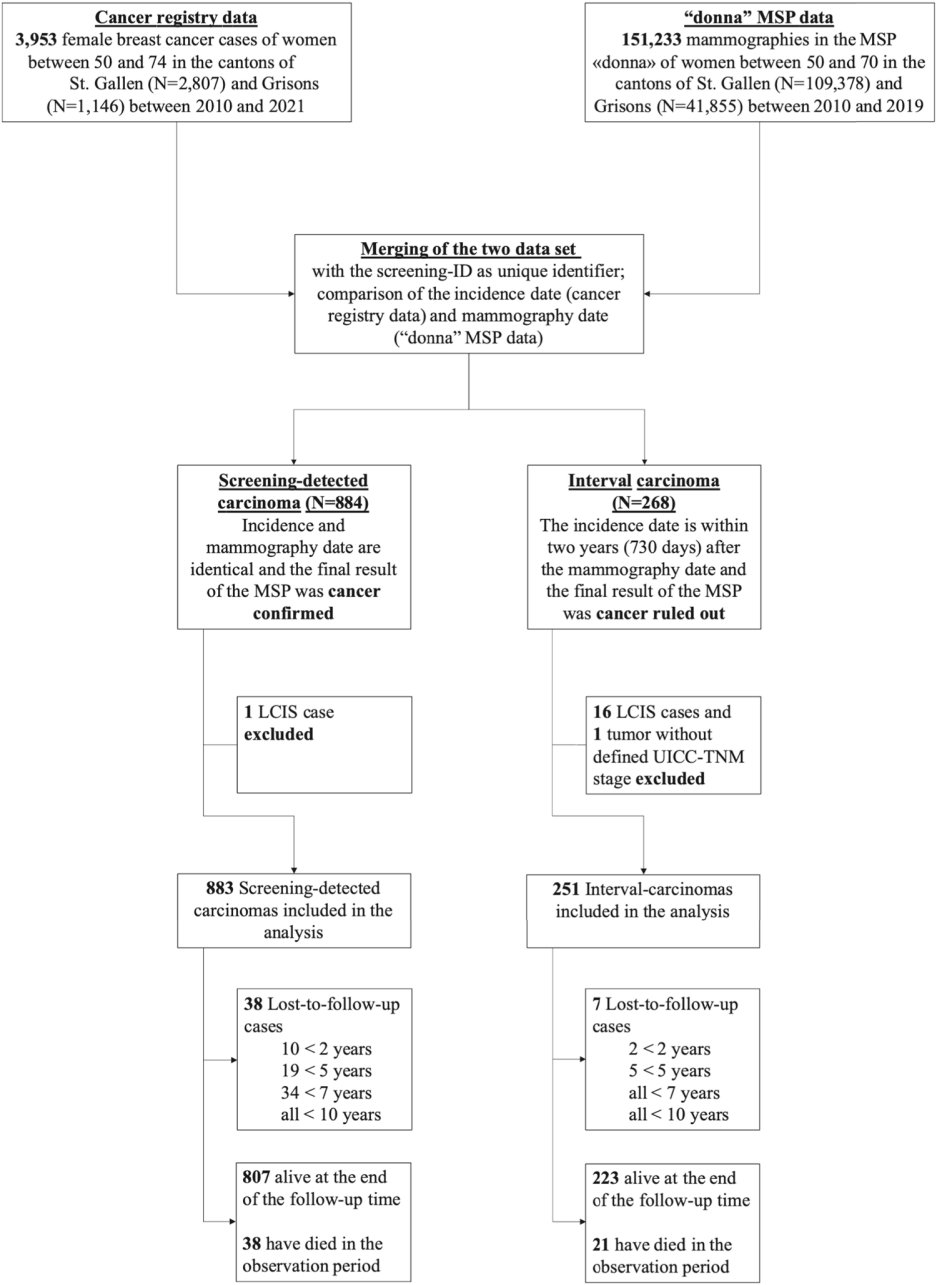
Derivation of the final data set**. **Lobular carcinoma in situ (LCIS) cases were excluded from the analyses as “LCIS is a benign condition and is not treated as a carcinoma” [22]; similar as Kuklinski et al. [10]. Cancer registry data were reviewed up to 71 years to identify IBC cases for women who participated in the MSP with up to 69 years

The data from the cancer registries and from the MSP were merged using a unique screening-ID (see Fig. 1). A recorded tumor was classified as SBC when it was diagnosed within 6 months after an abnormal screening mammogram finding or as IBC when a BC got diagnosed within 24 months after a normal screening mammogram finding (similar as in Niraula et al. [14] and the European guidelines [12]). The study followed the STROBE guideline for observational studies [19].

The final sample includes variables from the MSP and the cancer registries for all BC cases. MSP data include mostly systematically noted data such as year of screening, age of the screened women, number of mammograms within the MSP, breast density (BI-RADS a: almost entirely fatty breasts to d: extremely dense breasts), both radiologist ratings (BI-RADS 1 to 5), consensus conference conclusions (BC ruled out or further investigation), further conducted examinations (e.g. MRI), overall MSP outcome (BC detection or cancer ruled out), and self-declared information as family BC history (mother, sister or daughter diagnosed with BC). Cancer registry data include systematically noted data such as specific diagnosis (e.g. ICD-10, TNM classification), individual specific information (e.g. age, birthplace), cancer biology information (e.g. hormone receptor status, Ki-67 proliferation index), treatment information (e.g. Swiss classification of operations), medical follow-up information (e.g. remission or recurrence of BC), and vital status (incl. date of last contact and death cause). Based on the specific ICD-10 diagnosis available from the cancer registry, women with LCIS (i.e. D05.0) have been excluded from the analysis. Patients from the canton of St.Gallen were followed up until the end of October 2023, patients from the canton of Grisons until the end of August 2023.

### Statistical analyses

At first, patient and tumor characteristics are reported for all BC cases and stratified according to the IBC and SBC subgroups. Chi-squared tests or student’s *t* tests, as appropriate, were used to test for significance in differences in patient-level variables between the IBC and SBC groups, including tumor characteristics. Also, we descriptively outlined in which semester the IBC cases occurred to assess the temporal relevance. Specifically, we studied whether the IBC cases were diagnosed within 6 months after a normal screening mammogram (semester 1), after 7 to 12 months (semester 2), 13 to 18 (semester 3), or 19 to 24 months (semester 4). The overall survival of the two groups was compared using Kaplan–Meier curves. The log-rank test of equality was used to test the equality of the 5- and 7-year survival functions (overall mortality) of the IBC and SBC groups as a whole and for the breast density-specific subgroups. To identify risk factors for IBC diagnoses, a multivariable Poisson regression with robust errors model was calculated with IBC as outcome, compared to SBC as reference. The approach was determined based on the prevalence of 22.1% IBCs and the nonconvergence of a binary logistic regression [20, 21]. Here, screening-year was used as confounder, and screening age groups, birthplace, place of living, breast density, and family BC history were used as predictors. Results were considered significant at the 95% confidence level. All statistical analyses were performed in Stata 18.

## Results

Each year around 18,500 mammograms are performed within the MSP, with a participation rate of approximately 50% of all eligible women. From 2010 to 2019, 334,150 women have been invited for screening, of which 151,233 presented for screening. Ultimately, there were 937 cases of invasive breast cancer and 197 of non-invasive breast cancer (in situ), after removing cases due to missing information (i.e. UICC-TNM stage) and LCIS cases as “LCIS is a benign condition and is not treated as a carcinoma” [22]. Among those, 883 were SBC and 251 were IBC. Figure 1 shows the derivation of the final data sample.

From all BC cases among women who participated in the MSP between 2010 and 2019, 77.9% of all BC cases have been detected during screening and 22.1% as IBC. Excluding the initial screening round, the distribution of IBC and SBC cases has been stable within the MSP (see Appendix figure A1). Temporally, 63.3% of IBC cases were detected in months 13–24 after screening, almost twice as many as in the first 12 months after screening (36.7%). Specifically, 86 IBC cases were diagnosed 18 to 24 months after screening (S4), representing 139% more IBC cases than in the first 6 months after screening (S1; see Appendix figure A2).

**Fig. 2:**
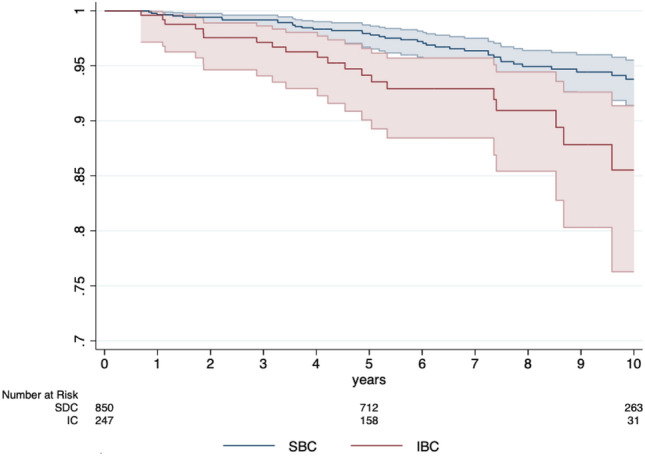
Kaplan–Meier curves of overall survival of women with IBC (red) vs SBC (blue) including 95% confidence interval**.** A second primary BC was either excluded if diagnosed after the first primary BC or if two BC had the same diagnosis date (i.e. 33 SBC and 4 IBC), the one with the more favorable tumor characteristics was excluded.

Table 1 outlines the patient and tumor characteristics of the two groups in focus (IBC vs SBC). We did not observe statistically significant differences in age at diagnosis, birthplace, and cantonal place of living between these two groups. Compared to women with SBC, women with IBC more often had a family history of BC (IBC: 27.1% vs SBC: 19.6%; *p* < 0.05), a higher breast density (BI-RADS c & d; IBC: 66.8% vs SBC: 51.1%; *p* < 0.05), and were diagnosed at higher tumor stages (stage III & IV; IBC: 22.3% vs SBC: 4.6%; *p* < 0.05). Of all IBC, 14.3% were diagnosed in stage III (vs 3.5% for SBC; *p* < 0.05) and 8.0% in stage IV (vs 1.1%; *p* < 0.05). IBC were on average diagnosed with larger tumor sizes compared to SBC (IBC: 23.4 mm vs SBC: 17.9 mm; *p* < 0.05) and were more likely to have axillary lymph nodes involvement at diagnosis (IBC: 26.5% vs SBC: 18.4%; *p* < 0.05). IBC also had higher histologic grade (grade III; IBC: 36.1% vs SBC: 19.7%; *p* < 0.05), were more often triple negative (i.e. estrogen- and progesterone-receptor negative and no HER2 overexpression; IBC: 8.0% vs SBC: 3.2%; *p* < 0.05), and had a higher Ki-67 proliferation index (i.e. ≥ 25%; IBC: 46.7% vs SBC: 30.0%; *p* < 0.05).

**Table 1:** Patient and tumor characteristics

	All observations		IBC		SBC		Test for differences IBC vs SBC
	*n*	Mean (SD) / Proportion	*n*	Mean (SD) / Proportion	*n*	Mean (SD) / Proportion	*p*-value
Persona data							
Age at diagnosis	1134	59.5 (6.3)	251	59.3 (5.7)	883	59.6 (6.4)	*p* = 0.52
Birthplace							*p* = 0.63
Outside Switzerland	285	25.1%	66	26.3%	219	24.8%	
In Switzerland	849	74.9%	185	73.7%	664	75.2%	
Canton							*p* = 0.33
Grisons	276	24.3%	67	26.7%	209	23.7%	
St.Gallen	858	75.7%	184	73.3%	674	76.3%	
Breast density (BI-RADS)							*p* < 0.05
BI-RADS a	70	6.2%	8	3.2%	62	7.0%	
BI-RADS b	445	39.3%	75	30.0%	370	41.9%	
BI-RADS c	581	51.3%	153	61.2%	428	48.5%	
BI-RADS d	37	3.3%	14	5.6%	23	2.6%	
Family BC history	241	21.3%	68	27.1%	173	19.6%	*p* < 0.05
MSP data							
Stage distribution							*p* < 0.05
In Situ	197	17.4%	20	8.0%	177	20.0%	
I	530	46.7%	83	33.1%	447	50.6%	
II	310	27.3%	92	36.7%	218	24.7%	
III	67	5.9%	36	14.3%	31	3.5%	
IV	30	2.6%	20	8.0%	10	1.1%	
Detection (after nth mammogram in MSP)							*p* < 0.05
1st mammography	638	56.4%	112	44.6%	526	59.7%	
2nd mammography	257	22.7%	82	32.7%	175	19.9%	
3rd mammography	157	13.9%	37	14.7%	120	13.6%	
4th mammography	80	7.1%	20	8.0%	60	6.8%	
Tumor characteristics							
Mean tumor size (mm)	1,084	19.1 (16.2)	231	23.4 (16.6)	853	17.9 (15.9)	*p* < 0.05
Tumor Size < 1 cm	270	24.9%	37	16.0%	233	27.3%	
Tumor Size < 2 cm	709	65.4%	110	47.6%	599	70.2%	
Tumor Size < 5 cm	1,033	95.3%	214	92.6%	819	96.0%	
Histologic grading							*p* < 0.05
Grade I	219	23.5%	35	15.4%	184	26.1%	
Grade II	492	52.8%	110	48.5%	382	54.2%	
Grade III	221	23.7%	82	36.1%	139	19.7%	
Lymph nodes involvement							*p* < 0.05
Positive (N1 +)	191	20.0%	49	26.5%	142	18.4%	
Negative (N0)	765	80.0%	136	73.5%	629	81.6%	
Hormone receptor							*p* < 0.05
Positive	937	91.8%	207	86.6%	730	93.4%	
Negative	84	8.2%	32	13.4%	52	6.6%	
Estrogen receptors							*p* < 0.05
Positive	1,048	92.4%	218	86.9%	830	94.0%	
Negative	86	7.6%	33	13.1%	53	6.0%	
Progesterone receptors							*p* < 0.05
Positive	920	81.1%	178	70.9%	742	84.0%	
Negative	214	18.9%	73	29.1%	141	16.0%	
HER2 over expression							*p* < 0.05
Positive	131	14.2%	45	19.7%	86	12.4%	
Negative	790	85.8%	184	80.3%	606	87.6%	
Triple negative	48	4.2%	20	8.0%	28	3.2%	*p* < 0.05
Ki-67 proliferation index							*p* < 0.05
Low (< 10%)	210	23.0%	41	18.1%	169	24.6%	
Medium (≥ 10, < 25%)	391	42.8%	80	35.2%	311	45.3%	
High (≥ 25%)	312	34.2%	106	46.7%	206	30.0%	

Cases might not sum up to the total 1134 BC cases due to missing values

The Kaplan–Meier curves (Fig. 2) highlight that for all stages, the survival proportions are continuously lower for IBC compared to SBC over the course of ten years. Specifically, the 5-year (94.1% IBC vs 97.9% SBC) and 7-year survival proportions (92.9% IBC vs 96.4% SBC) are significantly lower for IBC (see Table 2). When accounting for the favorable stage distribution of SBC compared to IBC, survival proportions for all stages besides the in situ stage are not statistically different (see Table 2).

**Table 2:** Comparative analyses of survival data

	All observations		Interval breast carcinoma (IBC)		Screen-detected breast carcinoma (SBC)		Log-rank test of equality
	*n*	Proportion %	*n*	Proportion %	*n*	Proportion %	*p*-value
Overall survival							*p* < 0.05
5-year	871	97.1	159	94.1	713	97.9	
7-year	629	95.6	102	92.9	528	96.4	
In situ							*p* < 0.05
5-year	163	98.9	14	94.4	150	99.4	
7-year	124	98.3	7	94.4	118	98.7	
Stage I							*p* = 0.90
5-year	408	98.7	52	97.1	357	99.0	
7-year	293	97.7	37	97.1	257	97.8	
Stage II							*p* = 0.54
5-year	245	98.3	64	100	182	97.6	
7-year	173	96.2	39	100	135	94.9	
Stage III							*p* = 0.79
5-year	47	91.9	24	90.2	24	93.4	
7-year	34	87.8	17	86.1	18	89.4	
Stage IV							*p* = 0.89
5-year	12	54.6	9	59.9	4	45.0	
7-year	9	49.7	6	52.4	4	45.0	

Second primary BC was either excluded if diagnosed after the first primary BC or if two BC had the same diagnosis date, the one with the more favorable tumor characteristics, based on UICC-TNM stage, T-Stage, histologic grading, tumor size, estrogen- or progesterone-receptor status, was excluded

The multivariable Poisson regression with robust errors (see Table 3) highlights three risk factors as statistically significant determinants of an IBC diagnosis in comparison to SBC detection: Age, breast density, and a family BC history.

**Table 3:** Multivariable Poisson regression with robust errors: Analysis of risk factors for an IBC diagnosis

Variable	Risk ratio (RR)	Confidence Interval (95%)
Screening age (years; reference: 50–54)		
vs 55–59	1.345*	1.027–1.761
vs 60–64	0.975	0.709–1.342
vs 65–69	0.721	0.513–1.013
Swiss vs foreign birthplace	0.990	0.772–1.270
St.Gallen vs Grisons place of living	0.895	0.702–1.141
Breast density (reference: BI-RADS a)		
vs BI-RADS b	1.367	0.702–2.661
vs BI-RADS c	2.093*	1.095–4.001
vs BI-RADS d	3.293**	1.542–7.031
Family BC history	1.299*	1.021–1.652
Constant	0.126**	0.041–0.387
Confounder: Screening-year (categorical); Significance levels: *p < 0.05; ** p < *0.01*		

Family BC history defined as mother, sister or daughter diagnosed with BC. The specific number/ proportion of women per sub-group exposed to each risk factor is seen in Table 1

The risks of getting diagnosed with an IBC (compared to SBC) were significantly higher with a high breast density compared to women with almost entirely fatty breasts (BI-RADS a). In particular, the risks (risk ratio: RR) were 2.1 times higher (*p* < 0.05) for women with heterogeneously dense breasts (BI-RADS c) and 3.3 times higher (*p* < 0.01) for women with extremely dense breasts (BI-RADS d). Further, women aged between 55 and 59 at last screening were significantly more likely (risk ratio: 1.3, *p* < 0.05) to get diagnosed with IBC compared to women between 50 and 54. Additionally, the risks of getting diagnosed with IBC were 1.3 times higher for women who have a family BC history (*p* < 0.05).

## Discussion

Our results found for a Swiss MSP that IBCs were diagnosed in more advanced stages compared to SBCs, were charactrized by a worse tumor biology (e.g. triple negative BC), and were associated with a worse overall survival. Further, high breast density (BI-RADS c & d), aged between 55 and 59, and a family BC history have been identified as significant risk factors for an IBC diagnosis.

When comparing the proportion of 22.1% IBC with 77.9% SBC with other national and international MSPs, the analyzed IBC proportion is at the lower end. Other MSPs reported a proportion of IBC cases between 21.8% and 31.2% [14, 16, 23–26]. The majority (63%) of IBC cases occurred within the second year after the screening, and especially within 18–24 months (S4) after the screening, as cancers develop and grow over time. Similar results have also been reported in other studies [25, 27–29]. Our findings confirm most previous studies, that the tumor biology of IBC compared to SBC is worse in all factors studied: higher tumor stages, larger tumor sizes, higher gradings, higher Ki-67 levels, and higher proportions of negative hormone receptors, triple negative tumors, and HER-2 overexpression [14–16].

Regarding the survival, the results indicated that the overall survival proportions for women with IBC from the MSP are significantly worse than those with SBC. This might largely be explained by the higher tumor stage(s) at diagnosis that we observed among those with IBC compared to SBC. When comparing the survival proportions of women whose IBC or SBC were detected at the same stage, we did not find statistically significant evidence for worse survival for most subgroups. The finding that IBC cases have worse survival proportions in general is consistent with other international studies [14, 30–33]. Hereby, multiple other studies similarly found that the survival differences are related to the worse tumor stage [28, 30], some other studies found statistically worse survival differences for IBC beyond these factors [14, 31]. Compared to women from the same region who were diagnosed with BC and did not participate in the MSP, women who did participate in the MSP and were diagnosed with BC had a significantly higher survival portion, even after adjusting for lead time and length bias [10].

We identified three potential risk factors for an IBC diagnosis: age, breast density, and a family BC history. We expected that women with heterogeneously and extremely dense breasts would have a higher risk of IBC given the greater challenge to identify BC signs in their mammography. But we did not expect certain age groups and women with a family BC history to have higher risks for getting diagnosed with IBC. In line with our findings, Ambinder et al. [15] found significant predictive relevance of a high breast density for an IBC diagnosis with an odds ratio of 2.17 for heterogeneously dense or extremely dense breasts. Similarly, Domingo et al. [34] found significant evidence with an odds ratio of 1.67 for extremely dense breasts for true IBC (excluding false negatives, minimal-sign cancers, and occult tumors). Thus, high breast density as risk factor for IBC is in line with the literature. Regarding age, Ambinder et al. [15] did not find any statistical relevance, but Kou et al. [16] showed that younger woman (< 50 years at diagnosis) had the highest odds (*p* < 0.01) to be diagnosed with IBC, similarly as Holm et al. [35], but this age group is not eligible to participate in the Swiss MSP “donna.” In absolute numbers, we also found the highest number of IBC cases in the youngest age group of the screening population (50–54 years). Risk-wise, we found that women between 55 and 59 had the highest likelihood of getting diagnosed with IBC compared to SBC. Regarding family BC history, while Kou et al. [16] only found some indication (*p* = 0.09) with increased odds of 1.92, Holm et al. [35] found significant evidence for family history of BC with an odds ratio of 1.32, which is in line with our findings. Additionally, multiple studies identified other risk factors that we did not have available (e.g. ethnicity) [15, 16].

### Limitations

The potential for residual confounding by unmeasured (e.g. ethnicity, socio-economic status [32]) or unrecognized factors because of observational data are inherent. Further, imperfect information (e.g. self-declared BC family history) and human error (e.g. false negative mammography reviews) might be present. Specifically, when retrospectively reviewing the original screening mammograms, Domingo et al. [34] identified that 23.6% of their IBC cases were false negatives where signs suspicious for malignancy can be seen retrospectively. Although our dataset is comprehensive as it includes all IBC cases from two Swiss cantons over ten years, the number of cases is limited (*n* = 251) and represents a relatively homogeneous Swiss population of women who were motivated to participate in the MSP [36].

The overall mortality rate of age-standardized BC has been declining over the last 30 years not only internationally, but also in our local data [37–39]. Simultaneously, the age-standardized incidence has been increasing [37, 38]. Improvements in treatment had an important and beneficial effect on BC mortality and might be more relevant for decreasing overall mortality than screening [40]. Using data of the two groups diagnosed and treated at the same time in the same area eliminates a bias caused by considerably better treatment regimens over time. Lead time bias and length bias have not been accounted for, as our analyses compare IBC and SBC, where both groups of women are regularly invited to participate in the MSP [41]. Furthermore, we had no information about the women at high risk based on the assessment of the individual doctors and their underlying criteria/ risk models, nor about the corresponding individualized solutions/ examinations.

## Conclusion

Overall, our results support the existing international evidence for the Swiss context that IBC differ in malignant tumor characteristics and have worse survival proportions than SBC due to more advanced stages. Further, based on the comprehensive long-term dataset, we were able to identify three relevant MSP-available risk factors for an IBC diagnosis that foster the current literature: Age, breast density, and family history of BC. While there is no systematic age trend visible, it is evident that women with higher breast density and women with a family BC history are of greater risk to get diagnosed with IBC.

Our findings highlight the relevance to reduce the incidence of IBC and have important practical implications for policy makers, MSP organizers, and participants. For instance, screening of women with known risk factors could be altered by, for example, utilizing other/or additional methods (e.g. MRI, contrast-enhanced mammography) or shorter screening intervals (e.g. annually) [42–44]. This represents a move toward more personalized screening strategies, which potentially increases screening quality and ideally also saves resources. Before implementing such personalized screening strategies, continued research is required, as data quality (e.g. breast density measured by software) and availability of potential risk factors require improvement. For example, high breast density (BI-RADS c & d) was identified as a significant risk factor, which underlines the importance of this factor. However, 44.8% of women in the MSP "donna" were assigned a high breast density, which is not precise enough for personalization yet. An (AI-based) risk model or a polygenic risk score could be a valuable addition for more personalized screening strategies to direct women to prevention strategies, which could then (potentially) reduce the risk of IBC [45–48].

## Figures and Tables

**Fig. 3:**
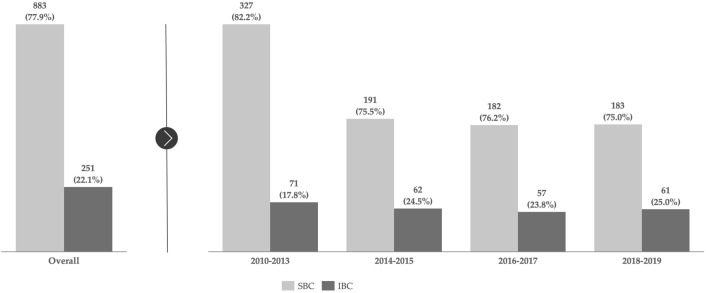
Cases of IBC and SBC in the MSP

**Fig. 4:**
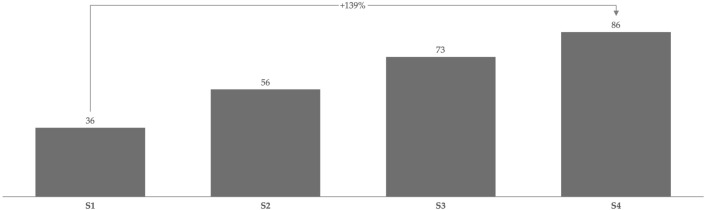
Timely distribution of IBC cases following screening (Note: S = Semester/ Half-year)

## Data Availability

Raw data for the mammography screening program “donna” and the Cancer Registry of Eastern Switzerland are not publicly available to preserve individuals’/patients′ privacy but pseudonymized data are available from the corresponding author on reasonable request.

## Appendix

See Figs. 3, 4.

## Abbreviations

BC: Breast carcinoma
IBC: Interval breast carcinoma
MSP: Mammography screening program
RR: Risk ratio
SBC: Screen-detected breast carcinoma
